# ERBB2-PTGS2 axis promotes intervertebral disc degeneration by regulating senescence of nucleus pulposus cells

**DOI:** 10.1186/s12891-023-06625-1

**Published:** 2023-06-20

**Authors:** Zhao-Cheng. Li, Fu. An

**Affiliations:** Department of Spine Surgery, Gansu Provincial Hospital of Traditional Chinese Medicine, Lanzhou, 730000 Gansu PR China

**Keywords:** Intervertebral disc degeneration, Senescence-related genes, ERBB2, PTGS2, Bioinformatics

## Abstract

Intervertebral disc degeneration (IDD) is considered one of the main causes of low back pain and lumbar disc herniation. Various studies have shown that disc cell senescence plays a critical role in this process. however, its role in IDD is yet unclear. In this study, we explored the role of senescence-related genes (SR-DEGs) and its underlying mechanism in IDD. A total of 1325 differentially expressed genes (DEGs) were identified using Gene Expression Omnibus (GEO) database GSE41883. 30 SR-DEGs were identified for further functional enrichment and pathway analysis, and two hub SR-DEGs (ERBB2 and PTGS2) were selected to construct transcription factor (TF)–gene interaction and TF-miRNA coregulatory networks, and 10 candidate drugs were screened for the treatment of IDD. Last but not least, in vitro experiments show that ERBB2 expression decreased and PTGS2 expression increased in human nucleus pulposus (NP) cell senescence model treated with TNF-α. After lentivirus-mediated overexpression of ERBB2, the expression of PTGS2 decreased and the senescence level of NP cells decreased. Overexpression of PTGS2 reversed the anti-senescence effects of ERBB2. The findings in this study suggested that ERBB2 overexpression further reduced NP cell senescence by inhibiting PTGS2 levels, which ultimately alleviated IDD. Taken together, our findings provide new insights into the roles of senescence-related genes in IDD and highlight a novel target of ERBB2-PTGS2 axis for therapeutic strategies.

**Supplementary Information:**

The online version contains supplementary material available at 10.1186/s12891-023-06625-1.

## Introduction

Intervertebral disc degeneration (IDD) is considered one of the main causes of low back pain and lumbar disc herniation [1]. As the world population continues to grow and age, the incidence of IDD will increase, resulting in a serious burden [2]. It has been reported that 84% of adults will experience low back pain in their lifetime, and approximately 23% will continue to experience work and life challenges associated with low back pain [3]. Until now, there are two primary treatment strategies for IDD: drug treatment and surgical interventions. Conservative drug treatment is preferred for early stage IDD while surgery is an effective method for treatment of advanced IDD. However, none of the current treatment measures can delay or reverse the progression of IDD [4]. Accumulating evidence suggests that different biological functions, such as apoptosis, senescence, and autophagy, play a role in IDD pathogenesis, among which cellular senescence plays a crucial role [5, 6].

Cellular senescence is defined as a permanent state of replicative arrest in otherwise proliferating cells. Changes in cell morphology include cell flattening, nuclear enlargement, and chromatin aggregation [7]. Another striking feature of senescent cells is that they secrete some chemokines, cytokines, growth factors, and matrix metalloproteinases (MMPs), which constitute the senescence-associated secretory phenotype (SASP) [8]. With increased aging, the continuous accumulation of senescent cells in IVD is the primary risk factor for chronic progression of IDD [9]. A growing body of evidence implicates a correlation between cellular senescence and pathogenesis of IDD. Related studies have shown that the number of senescent NP cells increases during IDD. SASP-mediated secretion by senescent cells accelerates the senescence of adjacent cells through autocrine and paracrine methods, which further reduces anabolism of the IVD extracellular matrix (ECM) and enhances catabolism, thereby accelerating IDD [10].

As a powerful inflammatory cytokine, TNF-α plays an important role in the process of inflammatory response in degenerative diseases [1]. TNF-α can aggravate the local inflammatory response in IVD by promoting the secretion of inflammatory factors such as COX-2 (cyclooxygenase 2), iNOS (inducible nitric oxide synthase), and IL-1β (interleukin-1β). Recent studies have shown that TNF-α plays an important role in the senescence of IVD cells [1, 7, 8]. However, the specific mechanism of TNF-α-mediated cellular senescence is still unclear.

**Fig. 1:**
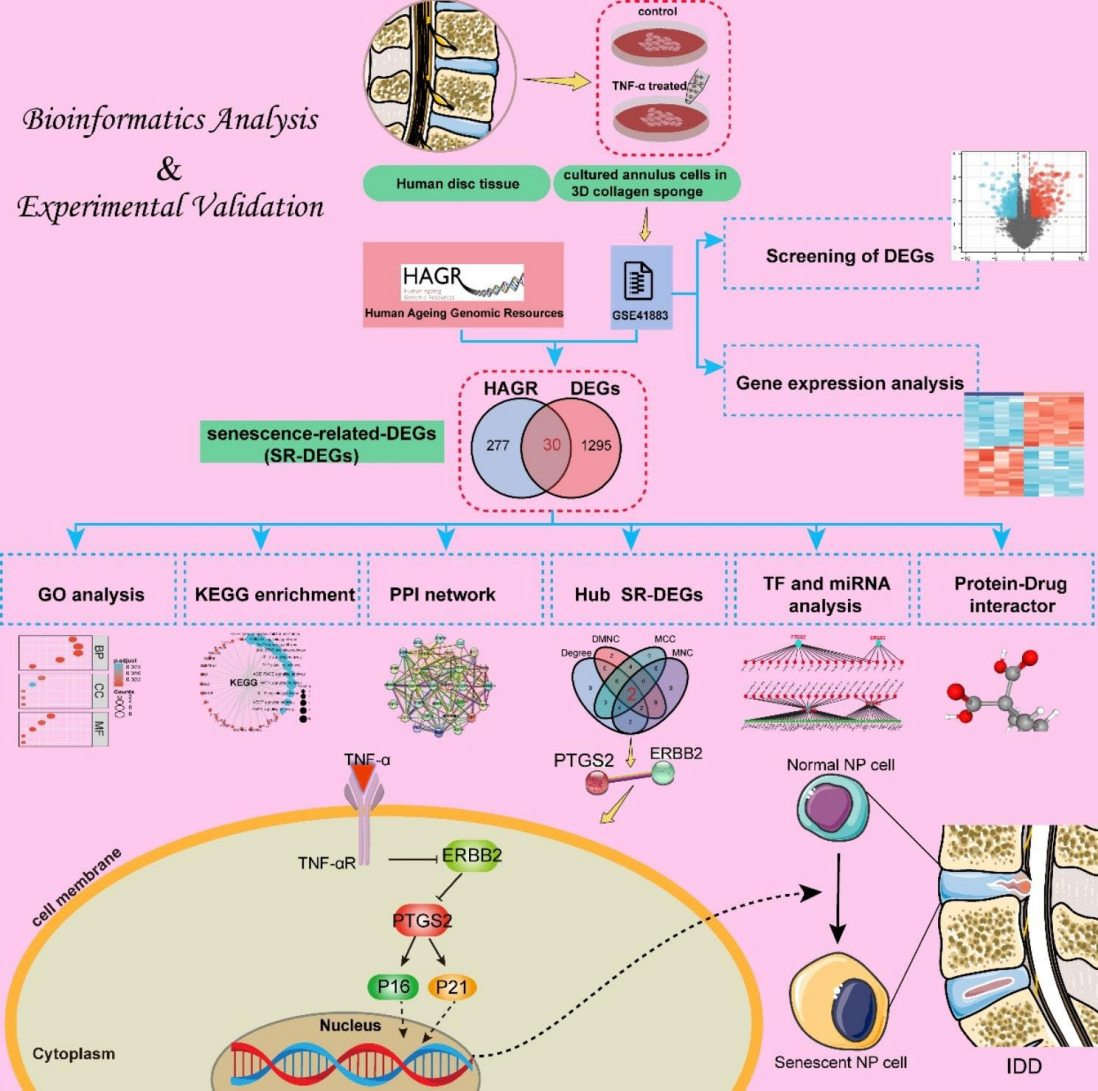
Flowchart of the present research

In recent years, bioinformatic analysis data of microarrays have been used to explore the mechanism of IDD, revealing some novel insights [11, 12]. Therefore, further mining of this high-throughput data will help reveal the underlying mechanism of IVD cell senescence. The aim of this study was to elucidate the underlying biological mechanism of senescence-related genes in IDD. First, we obtained the microarray dataset GSE41883 from the GEO database, and selected senescence-related-DEGs (SR-DEGs) from within the identified DEGs and performed function and pathway analysis of SR-DEGs. A protein–protein interaction (PPI) network of SR-DEGs was also established to screen hub SR-DEGs. Second, we predicted transcription factors (TFs) and microRNAs (miRNAs) that regulate hub SR-DEGs, as well as drug-targeting hub SR-DEGs. Finally, in vitro experiments show that ERBB2 expression decreased and PTGS2 expression increased in human NP cell senescence model treated with TNF-α, and ERBB2 overexpression further reduced NP cell senescence by inhibiting PTGS2 levels, which ultimately alleviated IDD. Our results yield insight into the role of senescence-related genes in IDD and highlight a novel target of ERBB2-PTGS2 axis for therapeutic strategies. A flowchart of the present research is displayed in Fig. 1.

## Materials and methods

### Data acquisition and processing

The GEO (http://www.ncbi.nlm.nih.gov/geo) database is a gene expression data access platform created and maintained by the National Center for Biotechnology Information (NCBI). It contains high-throughput gene sequencing and expression profiling data submitted by research institutions around the world. We downloaded and analyzed the gene profile dataset GSE41883 from the GEO database using the R software (ver. 3.6.3) GEOquery package [13]. The experimental design of GSE41883 data set is as follows: human disc tissue samples were obtained from patients with herniated discs and IDD disease. Cultured annulus cells were grown in a 3D collagen construct with or without 10e3 pM TNF-a for a total of 14 days. Following homogenization in TRIzol reagent, total RNA was isolated and analyzed via mircoarray. The source of the dataset is human, and the platform is based on the GPL1352 [U133_X3P] Affymetrix Human X3P Array. We used the affy package [14] to perform background correction and data normalization on the original CEL files of the GSE41883 dataset, and processed them in Bioconductor (http://www.bioconductor.org/) using the gene annotation file corresponding to the GPL1352 platform to annotate the probe matrix.

### Screening of DEGs and SR-DEGs

After data preprocessing, we used the limma package [15] to screen DEGs, with |log2 fold change (FC)| ≥ 1 and *P* < 0.05 as the cut-off criteria. We downloaded the senescence-related genes list from the Human Ageing Genomic Resources (HAGR) (https://genomics.senescence.info/). Then, senescence-related genes and DEGs were intersected to screen for SR-DEGs. At the same time, we analyzed the differential gene expression of SR-DEGs in the two groups of samples.

### Functional enrichment and pathway analysis of SR-DEGs

We performed GO and KEGG pathway enrichment analyses of SR-DEGs using the ClusterProfiler package [16, 17] in Bioconductor (http://bioconductor.org/packages/release/bioc/html/clusterProfer.html). *P* < 0.05 and count ≥ 2 were used as cut-off values, and the biological process (BP), cellular component (CC), molecular function (MF), and involved pathways of SR-DEGs were obtained. Finally, the results were visualized using the GOplot and ggplot2 packages.

### PPI network construction and identification of hub genes

STRING (https://cn.string-db.org/) was used as cellular PPI databases. [18]. We constructed the PPI network of SR-DEGs on STRING (ver. 3.9.0), set the biological species as “*Homo sapiens*” and the confidence as > 0.4 to obtain PPI network data, which was imported into Cytoscape (https://cytoscape.org/) for interaction network mapping and further analysis. We used the algorithms Degree, Maximum Neighborhood Component (MNC), Density of Maximum Neighborhood Component (DMNC), and Maximal Clique Centrality (MCC) of the cytoHubba plugin [19] in Cytoscape to screen the top 10 genes as hub genes. Hub SR-DEGs obtained by these algorithms were intersected, and a Venn diagram was generated.

### Transcription factor (TF)–gene interactions and TF–miRNA coregulatory network

Transcription factor (TF)–gene interactions with the identified hub SR-DEGs were determined to assess the regulatory role of TFs at the level of functional pathways and gene expression [20]. NetworkAnalyst (https://www.networkanalyst.ca/) was used to identify TF–gene interactions with the identified genes [21]. The TF–gene interaction network was generated by the JASPAR (https://jaspar.genereg.net/) database included in the NetworkAnalyst platform.

### TF–miRNA coregulatory network

TF-miRNA coregulatory interactions were collected from the RegNetwork repository [22], which facilitates the detection of miRNAs and regulatory TFs that regulate DEGs of interest at post-transcriptional and transcriptional levels. NetworkAnalyst is used to navigate datasets to identify biological features and functions that lead to valid biological hypotheses [23]; in this study, NetworkAnalyst was used to visualize the TF–miRNA coregulatory network.

### Identification of candidate drugs

Identification of protein–drug interactions is an important aspect in the screening of potential drugs for IDD. Based on our screened hub SR-DEGs, drug molecules were identified using the Drug Signatures Database (DSigDB), which was accessed through Enrichr (https://amp.pharm.mssm.edu/Enrichr/), a popular analysis platform with many gene set libraries for exploring genome-wide gene set enrichment [24, 25].

### Isolation and culture of human NP cells

According to the Pfirrmann grading system [26], grade II NP tissue samples (n = 3; 3 females; age 16–21 years, mean 18.33 years) were collected for NP cell extraction and qPCR assay. Grade V NP tissue samples (n = 3; 3 females; age 66–68 years, mean 66.66 years) were collected for qPCR assay. All the NP samples were from patients undergoing spinal surgery. All clinical tissue samples used in this study were obtained with patient consent and signed informed consent. All procedures were approved by the Ethics Committee of Gansu Provincial Hospital of Traditional Chinese Medicine. The NP tissue pieces were cut into pieces and placed in 0.25% trypsin-EDTA for digestion for 20 min; the supernatant was discarded, and the tissue pieces were removed. Transfer to 0.1% type II collagenase digestion solution, digest at 37 °C with constant temperature shaking for 30 min; after centrifugation, resuspend the bottom cell pellet with DMEM/F12 complete culture medium (containing 10% FBS), To be further cultured in vitro, the NP cells of 2–8 passages were used for in vitro experiments.

### Cell transfection

To create a ERBB2 and PTGS2 ectopic expression vector, the coding sequence (CDS) of ERBB2 and PTGS2 was inserted into the pLVX-Puro vector. We used the following primers to amplify the ERBB2 and PTGS2 CDS:

ERBB2-F 5′- ATGAAGCTGCGGCTCCCTGCCAGTC-3′;

ERBB2-R 3′- GACTGGCAGGGAGCCGCAGCTTCAT-5′;

PTGS2-F 5′- ATGCTCGCCCGCGCCCTGCTGCTGT-3′;

PTGS2-R 3′- ACAGCAGCAGGGCGCGGGCGAGCAT-5′. For transfection of 293T cells, the cells were cultured in 6-well plates, and then transfected with pLKO. Vectors were harvested after 48 h of transfection and were utilized to transduce NP cells.

### Cell immunofluorescence detection

The third-generation human NP cells was tested, remove the culture medium, wash twice with PBS buffer, incubate with 4% neutral paraformaldehyde fixative solution for 5 min at room temperature; wash twice with PBS buffer, 0.1% TritonX-100 cells were incubated with permeabilization solution at room temperature for 20 min; goat serum was blocked for 1 h; blocking solution was removed, Collagen II(1:200), Agreecan (1:200), ERBB2(1:150), PTGS2(1:200),and P16(1:200) (Affinity Biosciences, China) were added, and incubated overnight at 4 °C; primary antibody was removed and washed with PBS for 5 min×3 times, add fluorescent secondary antibody working solution and incubate for 1 h; discard the secondary antibody, wash with PBS for 5 min×3 times, add DAPI staining working solution and incubate for 20 min; observe under an inverted fluorescence microscope and take pictures for recording.

### Quantitative polymerase chain reaction (qPCR) assay

Total RNA from the chondrocytes was extracted using TRIzo (Invitrogen, Carlsbad, CA, USA) and was preserved at ‑80˚C. Complementary (c)DNA was synthesized from 1 µg total RNA using a cDNA synthesis kit (Takara Bio, Shiga, Japan). Reverse transcription‑quantitative PCR (RT‑qPCR) was performed using a cDNA reverse transcription kit (TaKaRa; Dalian, China). The PCR reaction conditions were as follows: pre-denaturation (95 °C for 30 s, 1 cycle), PCR reaction (95 °C for 5 s, 60 °C for 30 s, 40 cycles), dissolution (95 °C for 5 s, 60 °C for 1 min, 1 cycle) and Cooldown (50 °C for 30 s, 1 cycle). The primers for each gene were as follows:

PTGS2 forward, 5′‑AAAACTGCTCAACACCGGAA‑3′ and reverse, 5′‑GTGCACTGTGTTTGGAGTGG‑3′;

ERBB2 forward, 5′‑TTCCCTAAGGCTTTCAGTACC‑3′ and reverse, 5′‑TTCCCTAAGGCTTTCAGTACC‑3′;

GAPDH forward, 5′‑AATGGGCAGCCGTTAGGAAA‑3′ and reverse, 5′‑GCCCAATACGACCAAATCAGAG‑3′.

### Western blotting

NP cells were lysed in radioimmunoprecipitation assay buffer. Cell lysates were centrifuged at 13,000 rpm for 10 min at 4 °C and the supernatant was collected. Protein samples were separated by sodium dodecyl sulfate polyacrylamide gel electrophoresis (SDS-PAGE), and were then transferred onto polyvinylidene fluoride (PVDF) membranes. After blocking with 5% nonfat dry milk, the membrane was exposed to the primary antibody (ERBB2 (1:800), PTGS2 (1:800), P16 (1:1000), and P21 (1:1000) (Affinity Biosciences, China) and β-actin (1;5000) (ZSGB-Bio)) incubated at 4 °C overnight. Then membranes were incubated with the appropriate secondary antibodies for 1 h at room temperature. Protein bands were detected using an enhanced chemiluminescence kit and ImageJ software was used to process band intensities.

### Senescence-associated β-galactosidase (SA-β-gal) staining

According to the instructions of the manufacturer, SA–β-gal staining was done using a SA–β-gal staining kit (Beyotime, Shanghai, China) according to the manufacturer’s instructions.

### Alcian blue staining

The experiment was performed according to manufacturer’s instruction, the Alcian blue staining Kit (Beyotime, Shanghai, China) was used to assay proteoglycan.

### Statistical analysis

The results are presented as mean ± standard deviation, and data were analysed using the the GraphPad Prism software (ver. 8.0). Differences between groups were determined using Student’s *t-test* or one-way ANOVA. *P* < 0.05 was considered indicative of significance.

## Results

### Identification of DEGs and correlation analysis

**Fig. 2:**
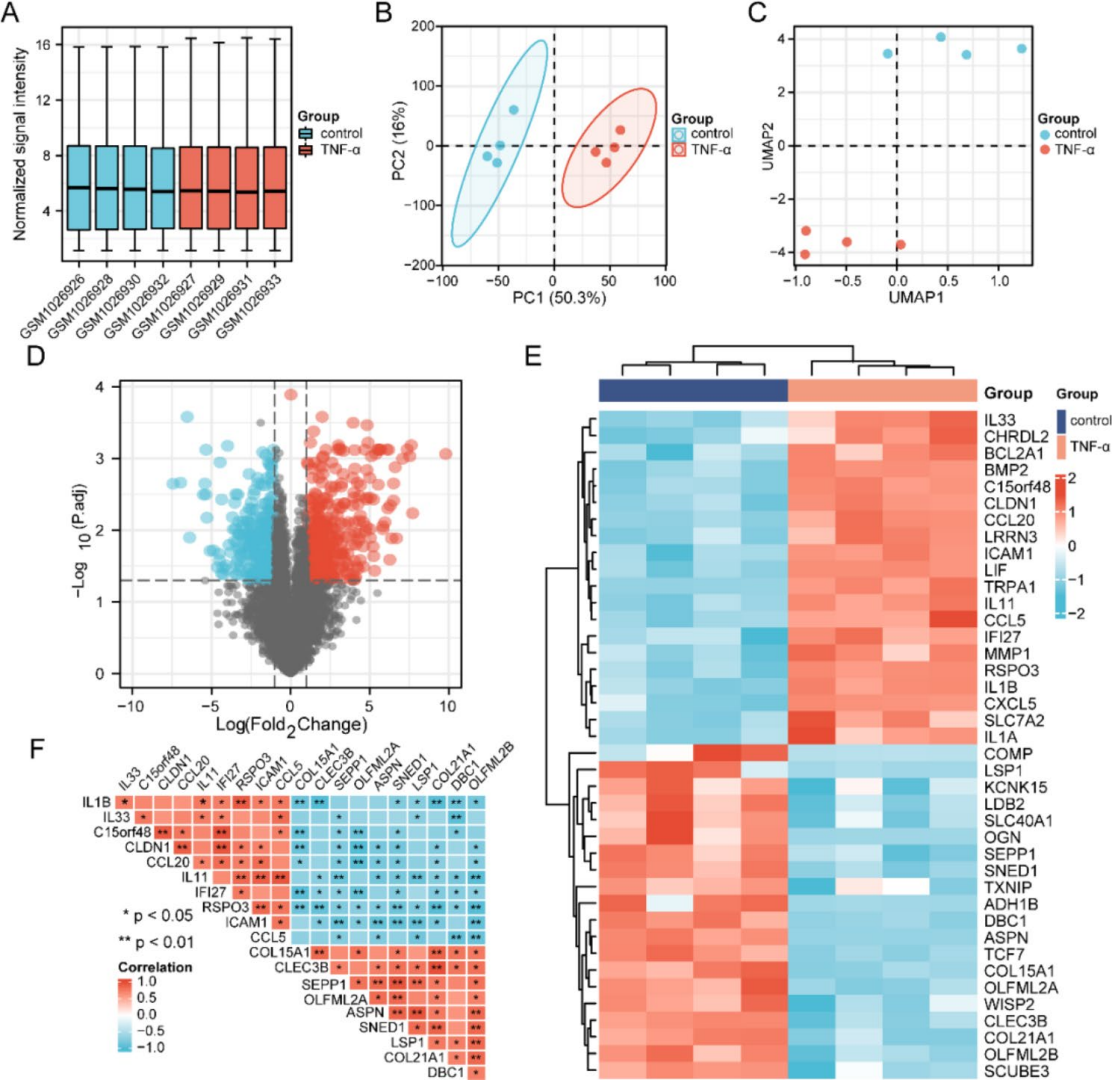
Identification of DEGs and correlation analysis. (**A**) Boxplots of sample expression levels after normalization of the GSE41883 dataset. (**B**) Principal component analysis (PCA) after normalization of the GSE41883 dataset. (**C**) UMAP analysis of GSE41883 dataset normalization. (**D**) Volcano plot of DEGs. Red indicates upregulation, blue indicates downregulation, and gray is used to represent the remainder. (**E**) Heat map of high- and low-expression top 20 DEGs. Red and blue represent upregulated and downregulated genes, respectively. (**F**) Bitmap of Pearson correlation analysis of the top 10 upregulated and downregulated DEGs. Red and blue represent positive and negative correlation, respectively

First, the GSE41883 dataset was standardized, and the results after data correction are displayed in box plots. The median of each sample was nearly horizontal, indicating the degree of normalization between samples is better than before normalization (Fig. 2A). In PCA principal component analysis and scatter plot display, each point in the scatter plot represents a sample, and the ratio of PC1 and PC2 was high, indicating obvious differences between groups (Fig. 2B). Uniform manifold approximation and projection (UMAP) analysis showed that the samples in each group were well separated, indicating significant differences between the sample groups, which was helpful for subsequent difference analysis (Fig. 2C).

In DEGs expression analysis, |logFC| ≥ 1 and *P* < 0.05 were used as cut-off values. A total of 1325 DEGs were screened, including 659 high-expressing and 667 low-expressing DEGs (Fig. 2D). The top 20 high and low expression DEGs were displayed in a heatmap; these genes were significantly different between the control and TNF-α treated group. The top three upregulated and downregulated genes were IL1B, IL33, and C15orf48, and COL15A1, CLEC3B, and SEPP1, respectively (Fig. 2E).

To clarify the correlation between DGEs, the top 10 up- or downregulated DEGs were selected for correlation analysis. The correlation analysis between DEGs is shown in a bitmap. As listed in Fig. 2F, IL1B was significantly positively correlated with RSPO3. However, IL1B was negatively correlated with COL15A1, CLEC3B, and COL21A1. CLDN1 had a significant positive correlation with CCL20 and IFI27, and a highly negative correlation with COL15A1 and OLFML2A1. In addition, a stronger negative correlation existed between CCL5 and DBC1. RSPO3, ICAM1, and CCL5 also showed relatively obvious positive correlations with IL11.

### Screening of SR-DEGs

**Fig. 3:**
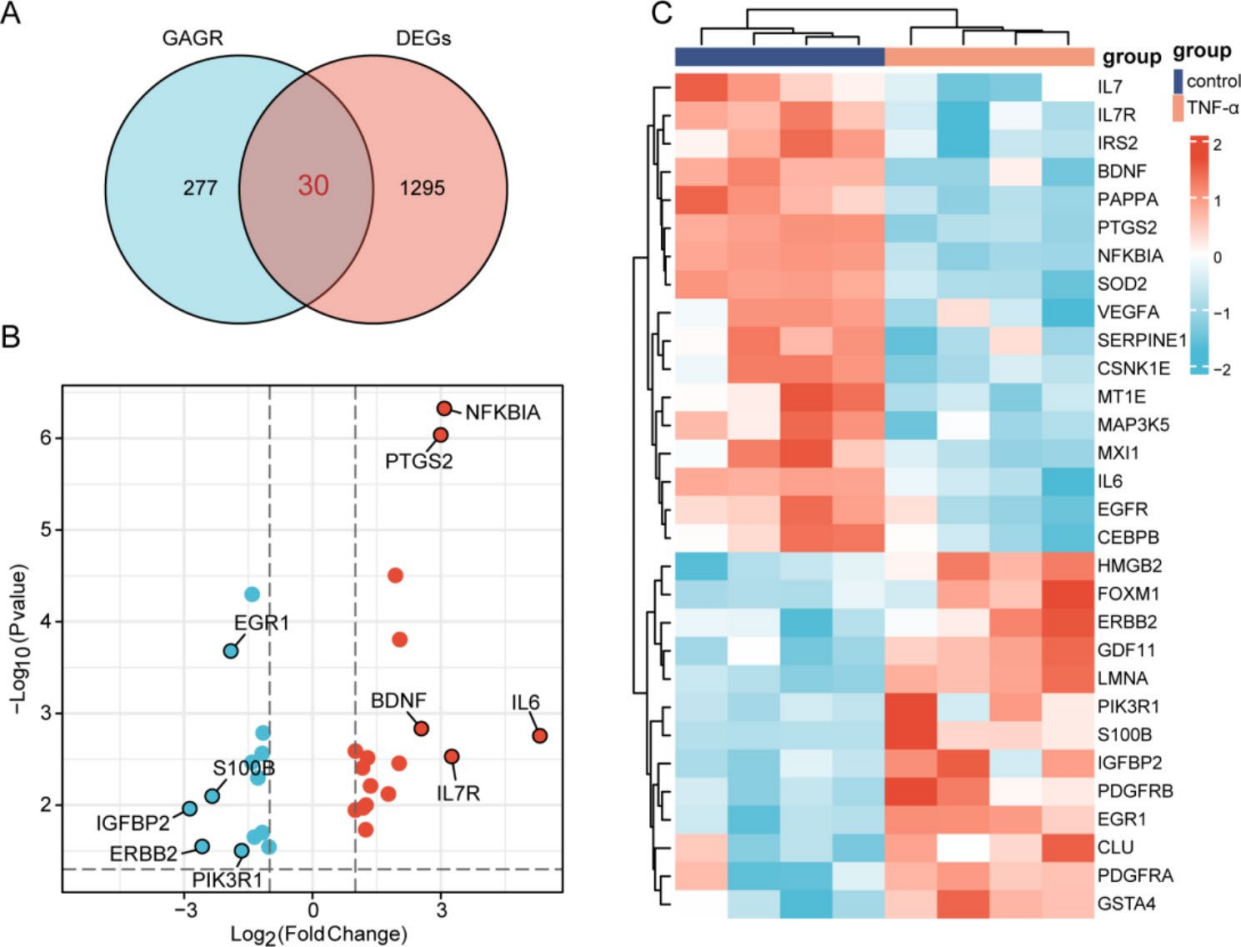
Screening of SR-DEGs. (**A**) Venn diagram showing 30 SR-DEGs selected by intersection of the senescence-related gene list and DEGs in the HAGR database. (**B**) Volcano plot of SR-DEGs in the control and TNF-α treated groups. (**C**) Heat map of 30 high- and low-expression SR-DEGs. Red and blue represent upregulated and downregulated genes, respectively

We screened SR-DEGs from the intersection of senescence-related genes and DEGs obtained from the Human Ageing Genomic Resources (HAGR). A total of 30 SR-DEGs were screened; 17 genes were upregulated and 13 downregulated in the TNF-α-treated group (Fig. 3A). The top five upregulated genes were IL6, IL7R, NFKBIA, PTGS2, and BDNF, and the top five downregulated genes were PIK3R1, EGR1, S100B, ERBB2, and IGFBP2 (Fig. 3B). Expression changes of 30 SR-DEGs and cluster analysis are displayed with a heat map (Fig. 3C).

### Functional enrichment and pathway analysis of SR-DEGs

**Fig. 4:**
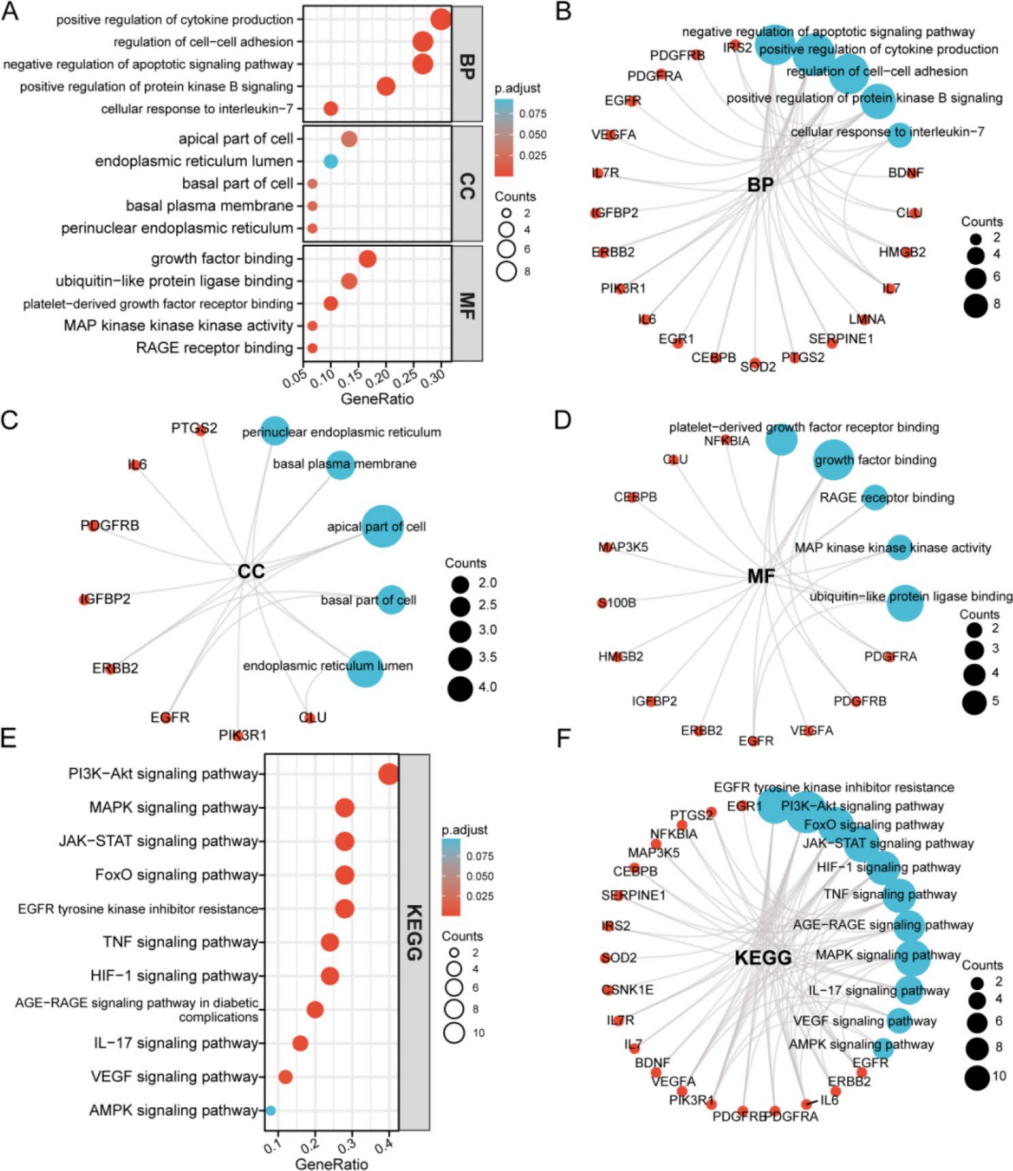
Functional enrichment and pathway analysis of SR-DEGs. (**A**) GO enrichment analysis of SR-DEGs. (**B**–**D**) Circle graph showing the SR-DEGs enriched in the different GO categories (BP, CC, and MF). The blue points represent the GO category, and the size of a point indicates the number of the genes it includes. (**E**) KEGG pathway enrichment analysis of DEGs. (**F**) Circle graph showing the number of SR-DEGs enriched in KEGG pathway analysis

To investigate the function of SR-DEGs, we performed GO and KEGG enrichment analyses. The GO terms consist of three parts: cellular component (CC), biological process (BP), and molecular function (MF). SR-DEGs of BP were involved in positive regulation of cytokine production, regulation of cell-cell adhesion, negative regulation of apoptotic signaling pathway, positive regulation of protein kinase B signaling, and cellular response to IL-7. CC analysis revealed that SR-DEGs were markedly enriched in the apical part of cell, endoplasmic reticulum lumen, basal part of cell, basal plasma membrane, and perinuclear endoplasmic reticulum. For MF analysis, the significantly enriched terms were growth factor binding, ubiquitin-like protein ligase binding, platelet-derived growth factor receptor binding, MAP kinase kinase kinase activity, and RAGE receptor binding (Fig. 4A; Table 1). The cnetplot function in the R software ClusterProfiler package was used to display enriched SR-DEGs in BP, CC, and MF (Fig. 4C–E).

**Table 1:** Functional enrichment analysis of SR-DEGs.

Ontology	ID	Description	GeneRatio	BgRatio	pvalue	p.adjust	qvalue
BP	GO:2,001,234	negative regulation of apoptotic signaling pathway	8/30	230/18,670	2.18e-09	4.29e-06	1.97e-06
BP	GO:0001819	positive regulation of cytokine production	9/30	464/18,670	3.01e-08	1.52e-05	6.98e-06
BP	GO:0022407	regulation of cell-cell adhesion	8/30	403/18,670	1.70e-07	2.72e-05	1.25e-05
BP	GO:0051897	positive regulation of protein kinase B signaling	6/30	176/18,670	3.17e-07	3.47e-05	1.59e-05
BP	GO:0098761	cellular response to interleukin-7	3/30	40/18,670	3.55e-05	6.74e-04	3.09e-04
CC	GO:0097038	perinuclear endoplasmic reticulum	2/30	22/19,717	5.07e-04	0.013	0.009
CC	GO:0009925	basal plasma membrane	2/30	34/19,717	0.001	0.020	0.014
CC	GO:0045177	apical part of cell	4/30	384/19,717	0.003	0.026	0.019
CC	GO:0045178	basal part of cell	2/30	51/19,717	0.003	0.026	0.019
CC	GO:0005788	endoplasmic reticulum lumen	3/30	309/19,717	0.011	0.092	0.066
MF	GO:0005161	platelet-derived growth factor receptor binding	3/30	15/17,697	1.97e-06	8.41e-05	4.94e-05
MF	GO:0019838	growth factor binding	5/30	137/17,697	3.15e-06	8.41e-05	4.94e-05
MF	GO:0050786	RAGE receptor binding	2/30	11/17,697	1.51e-04	0.001	7.62e-04
MF	GO:0004709	MAP kinase kinase kinase activity	2/30	26/17,697	8.80e-04	0.006	0.004
MF	GO:0044389	ubiquitin-like protein ligase binding	4/30	308/17,697	0.002	0.011	0.006

BP: biological process. CC: cellular component. MF: molecular function

The enrichKEGG function in the ClusterProfiler package was also used to enrich the signaling pathways involved in SR-DEGs. The enriched KEGG pathways included the PI3K/Akt signaling pathway, MAPK signaling pathway, JAK/STAT signaling pathway, FoxO signaling pathway, EGFR tyrosine kinase inhibitor resistance, TNF signaling pathway, HIF-1 signaling pathway, AGE/RAGE signaling pathway, IL-17 signaling pathway, VEGF signaling pathway, and AMPK signaling pathway (Fig. 4B; Table 2). The circle graph shows the enriched SR-DEGs in each pathway (Fig. 4F).

**Table 2:** Functional enrichment analysis of SR-DEGs.

Ontology	ID	Description	GeneRatio	BgRatio	pvalue	p.adjust	qvalue
KEGG	hsa01521	EGFR tyrosine kinase inhibitor resistance	7/25	79/8076	2.73e-09	4.53e-07	2.30e-07
KEGG	hsa04151	PI3K-Akt signaling pathway	10/25	354/8076	4.19e-08	3.48e-06	1.76e-06
KEGG	hsa04068	FoxO signaling pathway	7/25	131/8076	9.48e-08	5.25e-06	2.66e-06
KEGG	hsa04630	JAK-STAT signaling pathway	7/25	162/8076	4.07e-07	1.35e-05	6.86e-06
KEGG	hsa04066	HIF-1 signaling pathway	6/25	109/8076	7.57e-07	2.09e-05	1.06e-05
KEGG	hsa04668	TNF signaling pathway	6/25	112/8076	8.89e-07	2.11e-05	1.07e-05
KEGG	hsa04933	AGE-RAGE signaling pathway in diabetic complications	5/25	100/8076	1.15e-05	1.73e-04	8.80e-05
KEGG	hsa04010	MAPK signaling pathway	7/25	294/8076	2.16e-05	2.98e-04	1.51e-04
KEGG	hsa04657	IL-17 signaling pathway	4/25	94/8076	1.81e-04	0.002	8.00e-04
KEGG	hsa04370	VEGF signaling pathway	3/25	59/8076	7.60e-04	0.004	0.002
KEGG	hsa04152	AMPK signaling pathway	2/25	120/8076	0.053	0.097	0.049

KEGG: Kyoto Encyclopedia of Genes and Genomes

### PPI network construction and identification of hub SR-DEGs

**Fig. 5:**
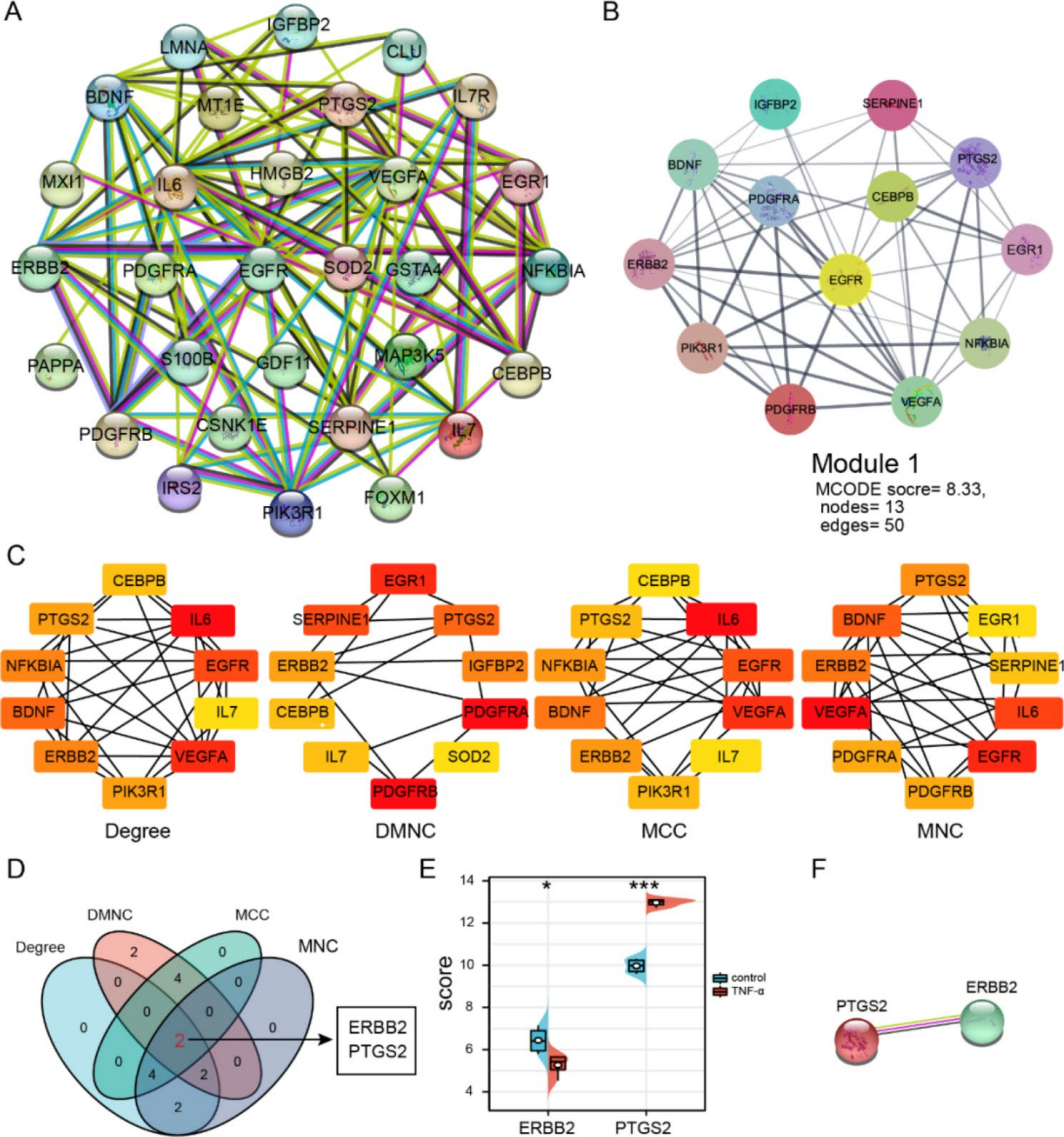
PPI network and module analysis. (**A**) The STRING database was used to construct a PPI network of SR-DEGs with 30 nodes and 100 edges. (**B**) Module 1 contained 13 gene nodes and 50 edges. MCODE score = 8.33. (**C**) Four algorithms (Degree, MNC, DMNC, and MCC) were used to obtain the top 10 genes as hub SR-DEGs. (**D**) Venn diagram showing the intersection of hub SR-DEGs obtained by four algorithms; ERBB2 and PTGS2 were finally obtained as hub SR-DEGs. (**E**) Analysis of two hub SR-DEG expression levels in two groups. (**F**) STRING database was used to construct the PPI network of two hub SR-DEGs.

To study the interactions between SR-DEG proteins, we constructed a PPI network of 30 SR-DEGs using the STRING database. The PPI network consisted of 30 nodes and 100 edges (Fig. 5A). Significant modules (score ≥ 4.5) were extracted from the PPI network. Module 1 contained 13 nodes and 50 edges (Fig. 5B).

We used the algorithms Degree, MNC, DMNC, and MCC of the cytoHubba plugin in Cytoscape to screen the top 10 genes as hub genes (Fig. 5C). Using the intersection of the hub genes obtained by the four algorithms, we obtained two hub genes, ERBB2 and PTGS2 (Fig. 5D). Compared to the control group, hub SR-DEG expression levels of ERBB2 were markedly downregulated while PTGS2 was markedly upregulated in the TNF-α-treated group (Fig. 5E). The STRING interaction network suggested an interaction between ERBB2 and PTGS2 (Fig. 5F).

### TF–gene interactions and TF–miRNA coregulatory network

**Fig. 6:**
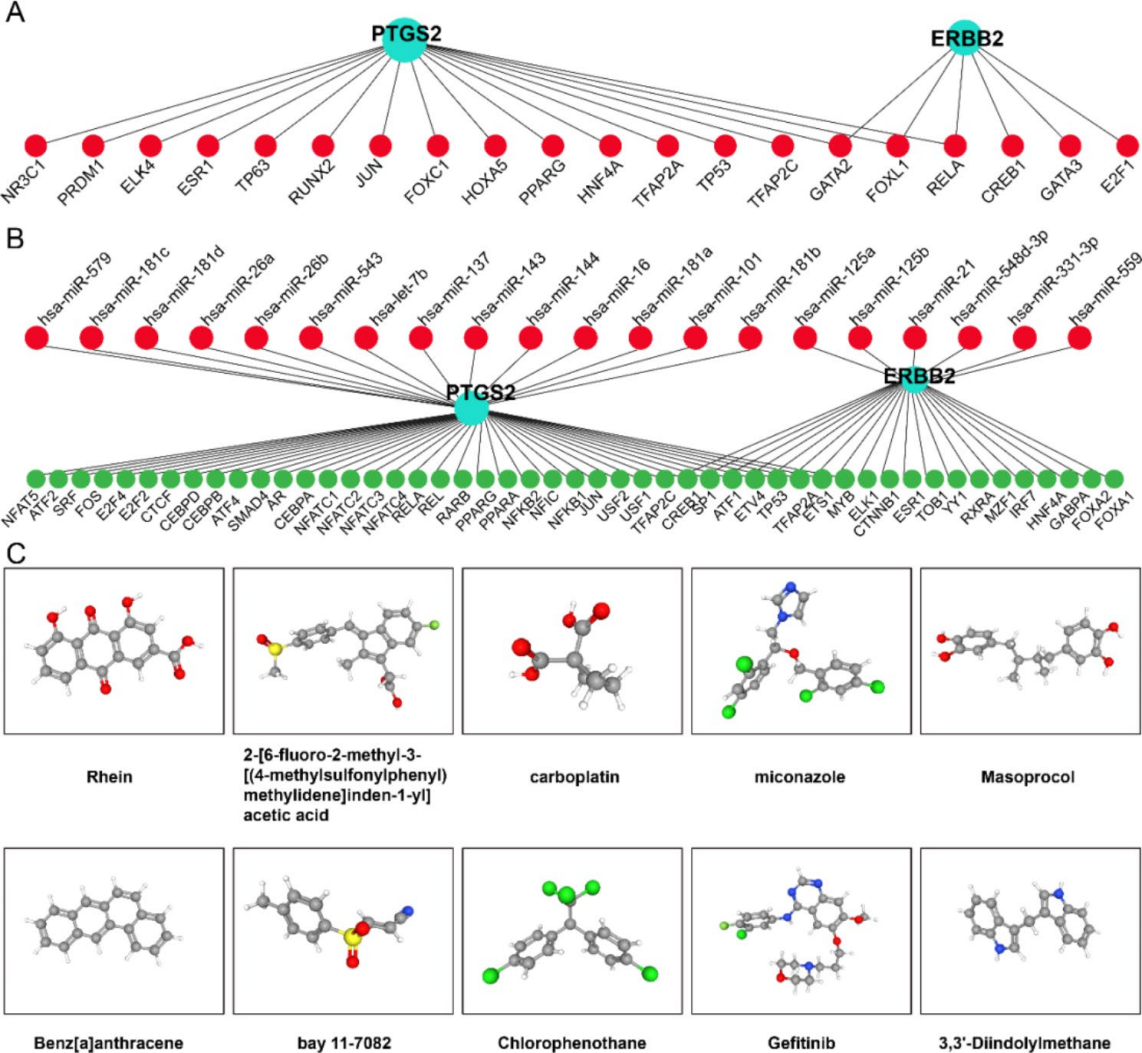
TF–gene interaction, TF–miRNA coregulatory networks, and Candidate drugs. (**A**) TF–gene interaction network with hub SR-DEGs. (**B**) TF–miRNA coregulatory network with hub SR-DEGs. (**C**) Candidate drugs for hub SR-DEGs from the DSigDB database

NetworkAnalyst was used to investigate the interaction between hub SR-DEGs (ERBB2 and PTGS2) and TFs. TF–gene interactions of ERBB2 and PTGS2 were identified, and interactions with hub SR-DEGs are visualized in Fig. 6A. The network contains a total of 20 TF–gene interactions with 22 nodes and 23 edges. PTGS2 is regulated by 17 TF genes, and ERBB2 is regulated by 6 TF genes. Among them, GATA2, FOXL1, and RELA can simultaneously regulate ERBB2 and PTGS2 (Fig. 6A).

The TF-miRNA coregulatory network was used to analyze the interactions of TF genes, miRNAs, and hub SR-DEGs, which may explain the changes in expression of the hub SR-DEGs. NetworkAnalyst was used to generate a TF–miRNA coregulatory interaction network with 20 miRNAs and 49 TFs interacting with hub SR-DEGs (Fig. 6B), with 71 nodes and 77 edges.

### Identification of candidate drugs

The Enrichr platform was used to identify potential drugs targeting hub SR-DEGs based on transcriptome signatures in the DSigDB database. According to *P*-value, the top 10 compounds were selected as candidate drugs. Table 3; Fig. 6C show candidate drugs for hub SR-DEGs obtained from the DSigDB database.

**Table 3:** List of the suggested drugs for IDD

Name	Chemical Formula	P-value	Rank
Rhein CTD 00001002	C15H8O6	4.7299E-06	1
2-[6-fluoro-2-methyl-3-[(4-methylsulfonylphenyl)methylidene]inden-1-yl]acetic acid CTD 00001194	C20H17FO4S	5.17489E-06	2
carboplatin CTD 00007106	C6H12N2O4Pt	5.63989E-06	3
miconazole	C18H14Cl4N2O	7.97987E-06	4
Masoprocol CTD 00006416	C18H22O4	1.07249E-05	5
Benz[a]anthracene CTD 00001470	C18H12	1.17299E-05	6
bay 11-7082 CTD 00003959	C10H9NO2S	1.70149E-05	7
Chlorophenothane CTD 00005755	C14H9Cl5	2.00249E-05	8
Gefitinib CTD 00003879	C22H24ClFN4O3	2.23249E-05	9
3,3’-Diindolylmethane CTD 00000841	C17H14N2	2.67799E-05	10

### Identification of human NP cells in culture

**Fig. 7:**
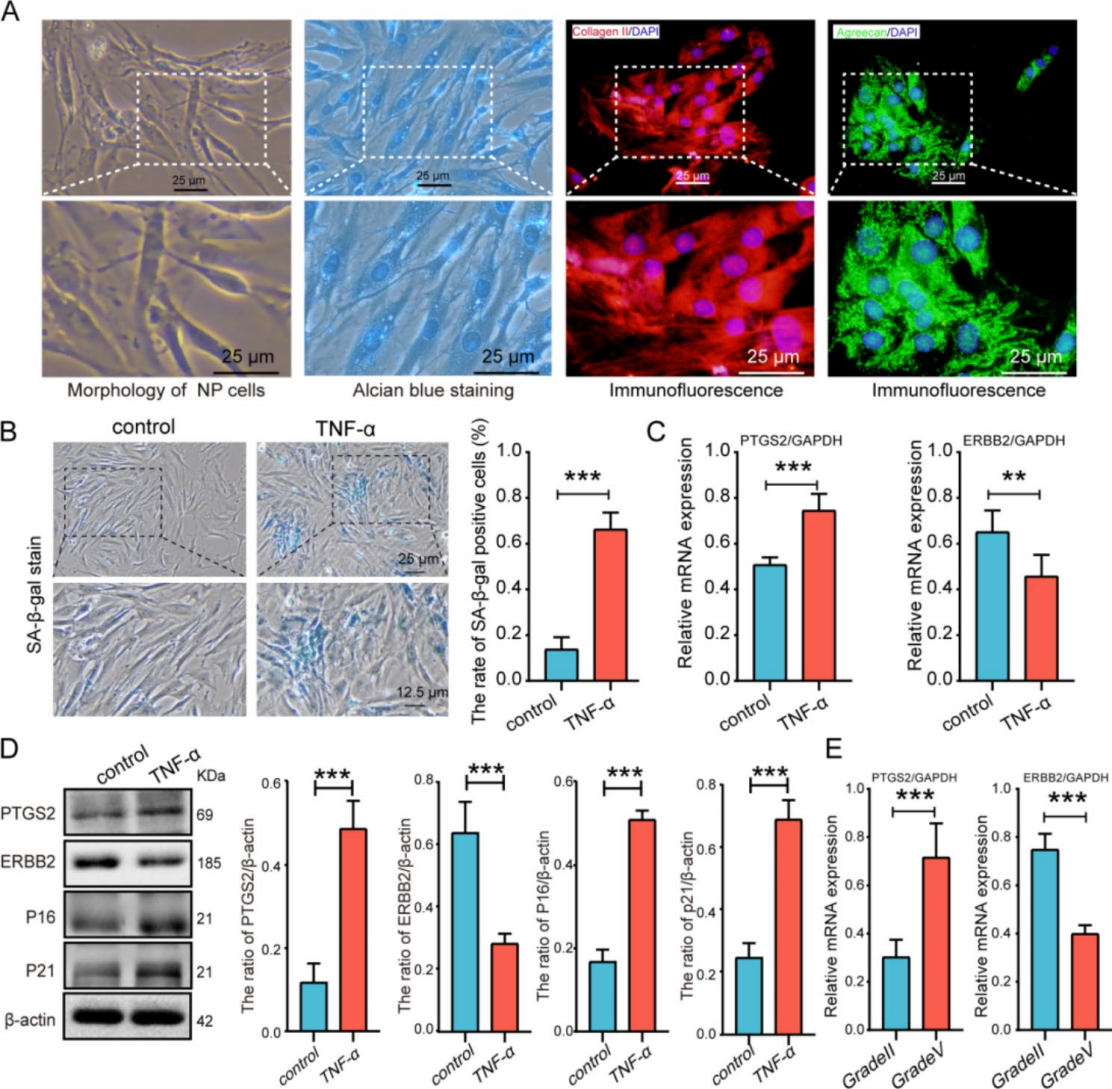
Identification of human NP cells in culture and the expression of PTGS2 and ERBB2 in senescent NP cells. (**A**) Identification of human NP cells. (Morphology of nucleus pulposus cells is mostly long fusiform, irregular or star shaped; The proteoglycans (PGs) released in the culture medium was measured using Alcian blue stain; collagen II and aggrecan fluorescence were detected). (**B**) SA-β-Gal staining assay of human NP cells. (**C**) qPCR showed that the PTGS2 and ERBB2 mRNA level in the treatment with or without TNF-α(20ng/ml,48 h). (**D**) Western blots and quantification of PTGS2, ERBB2, P16, and P21 in human NP cells after treatment with TNF-α(20ng/ml,48 h). (**E**) qPCR showed that the PTGS2 and ERBB2 mRNA level in the mild degenerated NP tissues (Grade II) and the severe degenerated NP tissues (Grade V). Quantitative measurements represent means ± SD (n = 3 biological replicates). Significant differences between the groups: **P < 0.01, ***P < 0.001

In addition, we extracted human primary intervertebral disc NP cells for further in vitro experiments. Alcian blue staining and immunofluorescence staining of collagen II and agreecan was used to identified NP cells. NP cell morphology is long spindle, triangular, irregular shape. Alcian blue staining showed rich proteoglycan expression in NP cells. Immunofluorescence staining showed extensive collagen II and aggrecan fluorescence in the cytoplasm, which increased with increasing proximity to the blue-stained nucleus (Fig. 7A).

### PTGS2 expression increased and ERBB2 expression decreased in senescent NP cells and degenerated NP tissues

The cellular degeneration model after TNF-α treatment is widely used to study IDD progression [27, 28]. After treatment of NP cells with TNF-α (20ng/ml, 48 h), SA-β-gal staining indicated that the level of cell senescence was significantly increased, and the results of qPCR indicated that the level of PTGS2 was increased, and the level of ERBB2 was decreased (Fig. 7B-C). Western blot indicated that the level of P16, P21, and PTGS2 was increased, and the level of ERBB2 was decreased (Fig. 7D). These results revealed that PTGS2 expression increased and ERBB2 expression decreased with NP cell senescence. In addition, we also examined the expression levels of PTGS2 and ERBB in degenerated NP tissues, and the results of qPCR indicated that the level of PTGS2 was increased, and the level of ERBB2 was decreased in the severe degenerated NP tissues (Grade V) (Fig. 7E).

### ERBB2 overexpression reduces TNF-α–induced NP cells senescence and the level of PTGS2

**Fig. 8:**
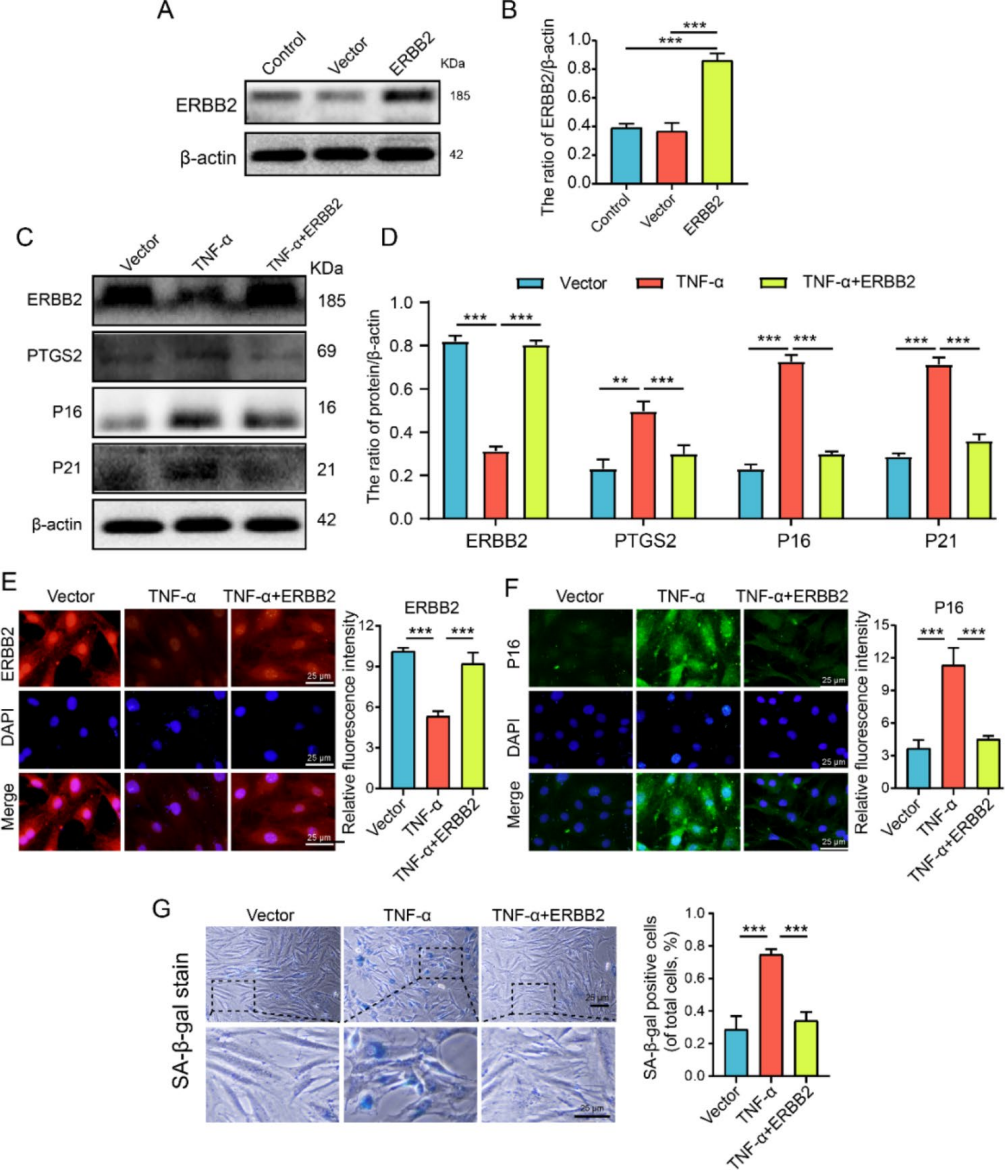
ERBB2 overexpression reduces TNF-α–induced NP cells senescence and the level of PTGS2. (**A**-**B**) Representative western blots and quantification of ERBB2 in the control, vector and ERBB2 groups. (**C**-**D**) Representative western blots and quantification of ERBB2, PTGS2, P16, and P21 in the vector, TNF-α and TNF-α + ERBB2 groups. (**E**-**F**) Representative immunofluorescence and quantification of ERBB2 (red) and P16 (green) in NP cells treated as above. (**G**) Representative SA-β-gal staining and quantification of SA-β-gal activity in the NP cells; blue, senescent NP cells. Quantitative measurements represent means ± SD (n = 3 biological replicates). Significant differences between the groups: **P < 0.01, ***P < 0.001

To clarify the changes in the senescence level of NP cells after ERBB2 overexpression, lentiviral overexpression system was used to increase the expression level of ERBB2 in NP cells, and western blot was used to verify the transfection efficiency. The transfection verification results showed that the transfection efficiency was over 80%, which met the experimental requirements (Fig. 8A-B). Western blot results showed that PTGS2, P16, and P21 were significantly increased in the TNF-α treated group compared with the vector group. The expressions of PTGS2, P16, and P21 were significantly decreased after ERBB2 overexpression (Fig. 8C-D). Results of immunofluorescence and SA-β-gal staining were consistent with those of western blot (Fig. 8E-G). These results indicate that the overexpression of ERBB2 significantly inhibits TNF-α-induced NP cells senescence, and this anti-NP cell senescence effect of ERBB2 may play a role by regulating the level of PTGS2.

### ERBB2 overexpression reduces senescence by decreases the level of PTGS2 in NP cells

**Fig. 9:**
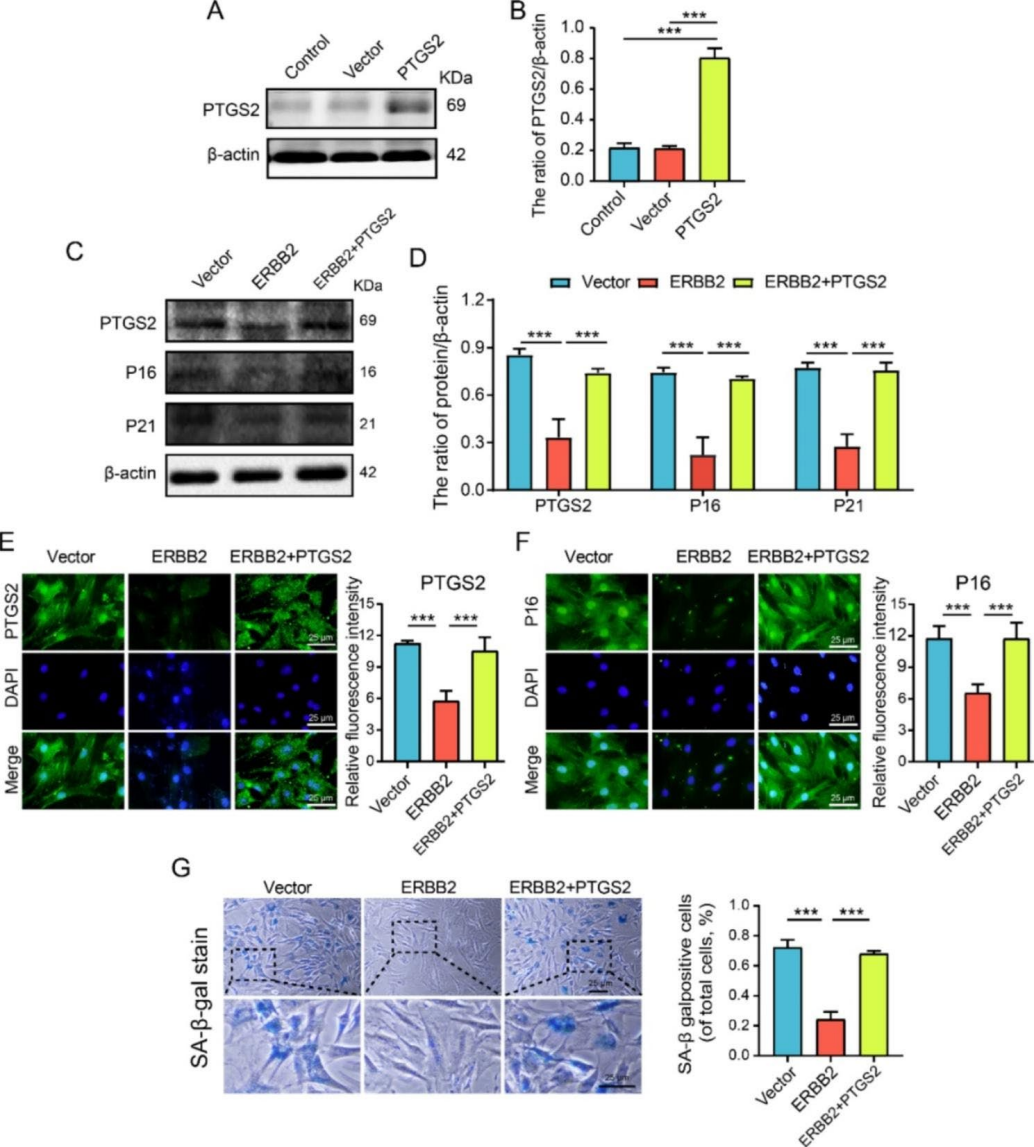
ERBB2 overexpression reduces senescence by decreases the level of PTGS2 in NP cells. (**A**-**B**) Representative western blots and quantification of PTGS2 in the control, vector and PTGS2 groups. (**C**-**D**) Representative western blots and quantification of PTGS2, P16, and P21 in the vector, ERBB2 and ERBB2 + PTGS2 groups. (**E**-**F**) Representative immunofluorescence and quantification of PTGS2 and P16 in NP cells treated as above. (**G**) Representative SA-β-gal staining and quantification of SA-β-gal activity in the NP cells; blue, senescent NP cells. Quantitative measurements represent means ± SD (n = 3 biological replicates). Significant differences between the groups: ***P < 0.001

In order to further clarify whether the anti-senescence effect of ERBB2 after overexpression is dependent on PTGS2, lentiviral overexpression system was used to increase the expression level of PTGS2 in NP cells, and western blot was used to verify the transfection efficiency. The transfection verification results showed that the transfection efficiency was over 80%, which met the experimental requirements (Fig. 9A-B). In TNF-α treatment, we overexpressed ERBB2 and simultaneously overexpressed PTGS2 to observe NP cell senescence. Western blot results showed that PTGS2, P16, and P21 decreased in the ERBB2 overexpression group compared with the vector group. In contrast to the ERBB2 group, the expression of P16 and P21 was enhanced after the simultaneous overexpression of ERBB2 and PTGS2 (Fig. 9C-D). Results of immunofluorescence and SA-β-gal staining were consistent with those of western blot (Fig. 9E-G). These results indicate that the overexpression of ERBB2 exerts the anti-senescence effect of nucleus pulposus cells by down-regulating the expression level of PTGS2.

## Discussion

IDD is a common spinal degenerative disease in orthopedics, and its incidence is positively correlated with age. Although many studies have revealed relevant mechanisms of IDD development, the underlying mechanisms are still not fully understood [6, 29]. In recent years, the role of cellular senescence in the occurrence and development of IDD has been more extensively investigated [30]. As a central inflammatory molecule, TNF-α is widely involved in IDD pathological processes, including cellular senescence, and anti-TNF-α therapy has shown great therapeutic promise in an in vitro IDD model [30]. In this study, we used the dataset GSE41883 to identify DEGs, which was combined with the HAGR database to screen SR-DEGs. We further performed functional enrichment analysis of SR-DEGs, PPI network construction, and identified hub SR-DEGs. We also identified TFs and miRNAs that regulate hub SR-DEGs, as well as drugs targeting hub SR-DEGs.

A total of 30 SR-DEGs were screened from the GEO and HAGR databases, including 17 upregulated and 13 downregulated genes. GO analysis revealed that SR-DEGs were markedly enriched in positive regulation of cytokine production, regulation of cell-cell adhesion, negative regulation of apoptotic signaling pathway, growth factor binding, platelet-derived growth factor receptor binding, and MAP kinase kinase kinase activity. Aging is responsible for the accumulation of senescent cells in IVD, a major risk factor for IDD progression [31]. Relevant studies have shown that the number of senescent nucleus pulposus cells increases significantly during IDD, and senescent cells can produce a large amount of cytokines, including pro-inflammatory cytokines (IL-1, IL-6, IL-8, and TNF-α), matrix-degrading proteases (MMPs and ADAMTS), growth factors (VEGF and TGFβ), and chemokines (CCL2 and CCL4), causing changes in ECM metabolism [32–35]. In recent years, the regulatory role of growth factors in IVD cell proliferation, apoptosis, and ECM production has attracted attention. Platelet-derived growth factor (PDGF) is a peptide modulator that stimulates tissue cell growth [36].

KEGG analyses were used to discover the main signaling pathways mediating IDD. The enriched KEGG pathways included the following: IL-17 signaling pathway, PI3K/Akt signaling pathway, JAK/STAT signaling pathway, FoxO signaling pathway, AGE/RAGE signaling pathway, and AMPK signaling pathway. During IDD progression, cytokines trigger Th17 cells to increase IL-17 production locally in IVD [37, 38]. The JAK/STAT and PI3K/AKT pathways are important signaling pathways for IL-17-mediated IDD. IL-17 activates the JAK/STAT pathway by mediating rapid tyrosine phosphorylation of the JAK family and STAT while activation of JAK by IL-17 induces activation of the PI3K/AKT pathway [16, 38]. He et al. [39] showed that IL-17 further induced the expression of Bcl-2 in nucleus pulposus cells by activating the PI3K/AKT pathway, thereby inhibiting autophagy. IL-23 is a pro-inflammatory cytokine in the IL-12 family, and its expression levels are significantly increased following disc herniation [40]. Gruber et al. [41] showed that human disc cells exposed to TNF-α exhibited significant upregulation of IL-23. Oxidative stress and inflammatory responses mediated by the AGE/RAGE signaling pathway play an important role in the progression of IDD [42]. Illien-Jünger et al. [43] suggested that activation of the AGE/RAGE pathway induces hypertrophy and osteogenic differentiation of IVD cells. More importantly, the accumulation of AGEs causes changes in the oxidative microenvironment of IVD, which in turn leads to IDD.

FOXO proteins are an evolutionarily conserved family of transcription factors with important functions in development, aging, and longevity [44]. In mammals, the FOXO family has four members (FOXO1, FOXO3, FOXO4, and FOXO6), of which FOXO6 is only expressed in neuronal tissues [45]. Alvarez-Garcia et al. [46] showed that levels of FOXO1, FOXO3, and FOXO4 were significantly reduced in degenerated IVD. AMPK is a highly conserved metabolic regulator in evolution [47]. Multiple studies have shown that AMPK is closely related to cellular senescence and aging-related diseases [48–50].

PPI analysis was used to identify hub SR-DEGs in IDD. We identified two SR-DEGs, ERBB2 and PTGS2. ERBB family receptors can recruit Shc and Grb2 proteins through their own phosphorylation sites to activate the RAS-RAF-MEK-ERK pathway, regulating cell proliferation, differentiation, and migration [51–53]. ERBB2 is a member of the ERBB family, which is located on chromosome 17q12 and encodes a transmembrane protein with a molecular weight of 185 kD and tyrosine kinase activity. ERBB2 was originally discovered in rat neuroblastoma, and point mutations in the transmembrane region can confer oncogenic activity [54]. In recent years, increasing evidence has supported the role of the ERBB2 gene in the progression of many human degenerative diseases, including IDD. Wang et al. [55] showed that the expression level of NRG1 was significantly reduced with IDD, while NRG1 overexpression increased the ratio of p-ERBB2/ERBB2 and decreased apoptosis of nucleus pulposus cells. Guo et al.[56]showed that upregulating the expression of ERBB2 in nucleus pulposus cells is beneficial to their survival under nutrient-deficient conditions, while downregulation of ERBB2 leads to a decrease in the phosphorylation of Erk1/2, which in turn promotes the development of IDD [57]. PTGS2, also known as COX-2, is a rate-limiting enzyme in the production of prostaglandin (PG) and a key regulator of inflammation [58]. Neidlinger-Wilke et al. [59] showed that the mesenchymal stem cell secretome decreases levels of inflammatory markers (PTGS2, IL-6, and IL-8) in annulus fibrosus organ cultures. Li et al. [60] identified PTGS2 as a key gene for IDD through mRNA sequencing studies.

Many studies have shown that TFs and miRNAs are important for understanding disease development. TFs are a class of sequence-specific DNA-binding proteins that regulate the rate and process of transcription of genetic information from DNA to RNA [61]. miRNAs play key roles in gene regulation and RNA silencing at the post-transcriptional level [62]. In this study, we revealed the potential relationship between hub SR-DEGs, TFs, and miRNAs. Among the TFs identified, ESR1 [63], RUNX2 [64], JUN [65], TFAP2A [66], TP53 [67], TFAP2C [68], CREB1 [69], and E2F1 [84] are closely linked to IDD. Among the miRNAs identified, hsa-miR-181c is involved in IDD progression [70]. In other disease studies, hsa-miR-26b [71] and hsa-miR-101 [72] have been shown to be targets of PTGS2 while hsa-miR-125a and hsa-miR-125b are targets of ERBB2 [73, 74].

The use of drugs in the early stages of IDD may be an effective means of treatment. Commonly used drugs include NSAIDs, which mainly relieve pain, but have many limitations and side effects. Pathological processes such as oxidative stress, the inflammatory response, and cellular senescence are closely related to IDD [1]. In this study, we screened 10 drugs for the treatment of IDD, which have shown great therapeutic potential in anti-cellular aging, oxidative stress, and the inflammatory response. Rhein, an anthraquinone molecule, can promote the synthesis of matrix components and inhibit the inflammatory response, thereby preventing IDD. Blocking lipopolysaccharide-induced NF-κB activation and cytokine production by Bay 11-7082 rescues premature senescence in adipocyte progenitors [75]. In tumor patients, pro-inflammatory cytokines can induce cellular senescence by activating EGFR signaling; this is inhibited by Gefitinib, a small-molecule inhibitor of EGFR [76]. Miconazole is an antimycotic drug that belongs to the azole family [77]. Previous studies have suggested that miconazole can activate the Nrf2 pathway to induce anti-oxidative stress and the inflammatory response, and has a protective effect on cells [78, 79]. These drugs may be promising candidates for the treatment of IDD.

However, there are still some limitations to our study. Firstly, due to the lack of IDD data sets with sufficient sample size, different data sets cannot be used to verify our analysis results. Secondly, although changes in PTGS2 and ERBB2 expression and their regulatory relationships were evaluated at the gene and protein levels, upstream TFs, miRNAs, and potential drugs were not validated. In future studies, we will further study the role of these PTGS2 and ERBB2 in the occurrence and development of IDD.

In conclusion, we identified 30 SR-DEGs using bioinformatics methods based on the data set GSE41883 and senescence-related genes in the HAGR database. Then, we constructed a PPI network using the 30 SR-DEGs and identified two IDD-related hub SR-DEGs (ERBB2 and PTGS2). TFs and miRNAs regulating hub SR-DEGs were analyzed by NetworkAnalyst, and we used the DSigDB database to identify possible novel drug molecules and drug-target interactions. Finally, in vitro experiments show that ERBB2 overexpression further reduced NP cell senescence by inhibiting PTGS2 levels, which ultimately alleviated IDD. Overall, the present study provides new insight into the pathogenesis and treatment of IDD.

## Electronic supplementary material

Below is the link to the electronic supplementary material.

Supplementary Material 1

## Data Availability

The GSE41883 datasets used in this study can be found in the Gene Expression Omnibus (https://www.ncbi.nlm.nih.gov/geo/). Other experimental data will be available upon request to the corresponding author.
