# Supplementary figures and images for: ERBB2-PTGS2 axis promotes intervertebral disc degeneration by regulating senescence of nucleus pulposus cells
Source: BMC Musculoskelet Disord. 2023 Jun 20;24:504. doi: 10.1186/s12891-023-06625-1 (PMC10280935; doi:10.1186/s12891-023-06625-1)

Figure 7

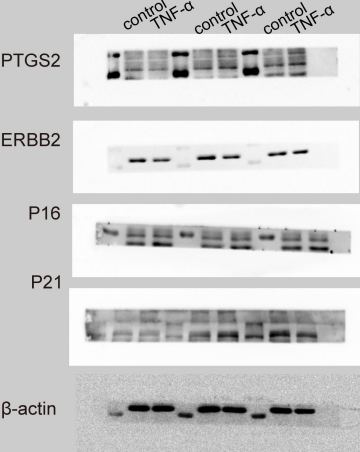

Figure 8

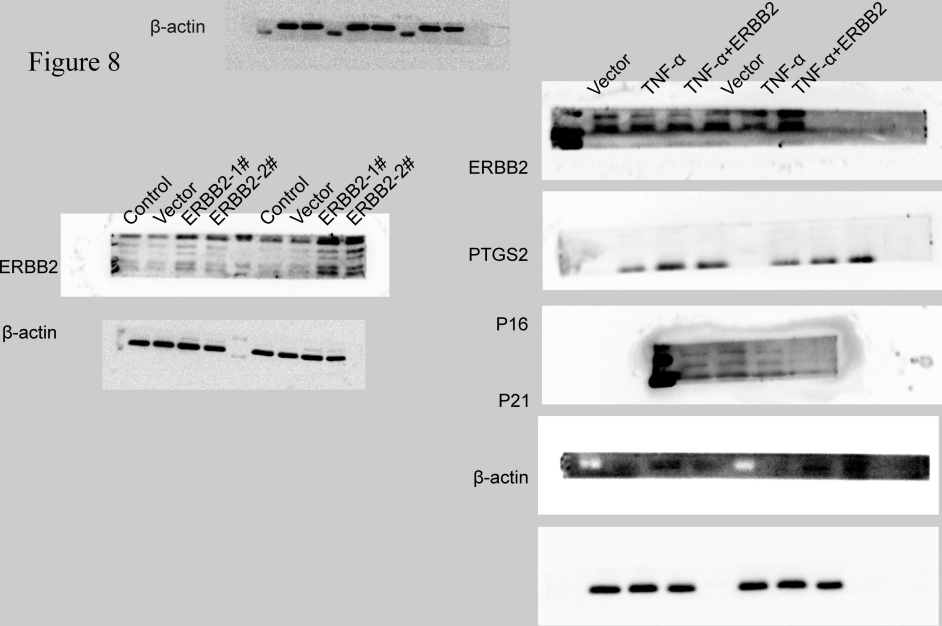

Figure 9

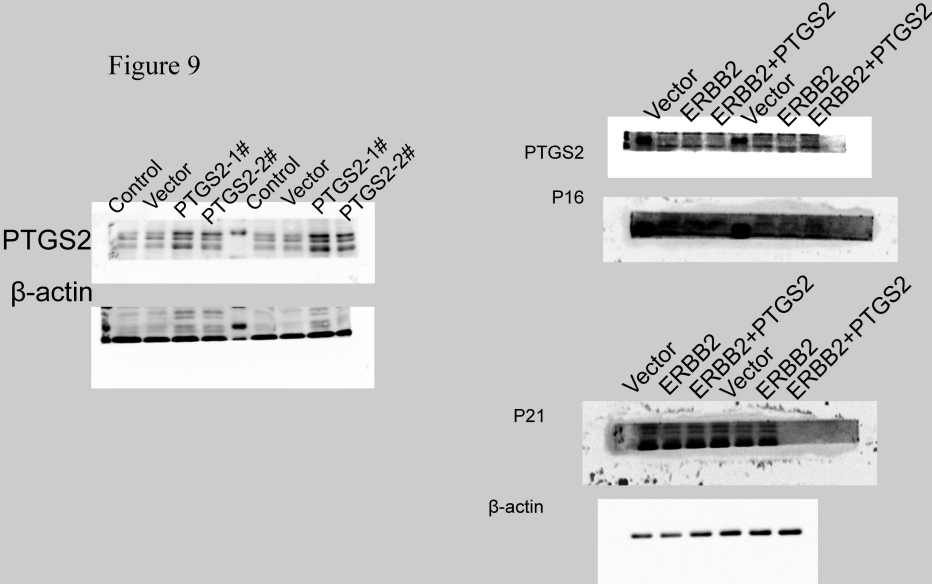

Supplement: Supplementary file 1 — Supplementary Material 1 [file 12891_2023_6625_MOESM1_ESM.pdf]
